# Turning acne on/off via mTORC1

**DOI:** 10.1111/exd.12180

**Published:** 2013-06-25

**Authors:** F William Danby

**Affiliations:** Department of Surgery, Section of Dermatology, Geisel School of Medicine at DartmouthHanover, NH, USA

**Keywords:** acne, dairy, diet, FoxO1, mTORC1, therapy

## Abstract

Over the past 10 years, the increase in comprehension of the mechanisms behind acne has been truly exponential. Starting with the ethnological work of Cordain, accelerated by the epidemiological work of Adebamowo, supported by the clinical trials of Smith and Mann, Kwon, DiLandro and others, the interface of diet and acne is coming into focus. Melnik now presents an exceptional pair of papers that illustrate for dermatologists what translational research is all about. The Western diet, the role of dairy, FoxO1 and mTORC1, the interplay of agonists and antagonists, therapeutics present and future – the jigsaw puzzle is coming together.

While it is distinctly unusual to have the pathogenesis of a disorder and the explanation for the efficacy of its treatments served up as a ‘fait accompli’, it is unheard of to have the entire picture knit together by one individual. But that is what Bodo Melnik may well have accomplished in two recent companion papers published in this journal, entitled ‘Potential role of FoxO1 and mTORC1 in the pathogenesis of Western diet-induced acne’ 1 and ‘Are therapeutic effects of anti-acne agents mediated by the activation of FoxO1 and inhibition of mTORC1 2?’

These scholarly articles might be retitled ‘Turning Acne On’ 1 and ‘Turning Acne Off’ 2. Melnik traces the impact of the high glycemic load Western diet as introduced to the discussion by Cordain 3 and the impact of dairy products as documented by Adebamowo 4–6, Smith and Mann 7–10, Kwon 11 and DiLandro 12.

The short story of the Western diet is that highly refined carbohydrates like white flour are easily converted to simple sugars. This diet also contains a generally increased sugar load and these influences combine to support a chronically increased plasma glucose level. The result is a low-grade hyperinsulinemia that, through a phosphorylation process, persuades the transcription factor FoxO1 to leave the cell nucleus, exposing the androgen receptor to circulating androgens 13.

The insulin level is also pushed higher by ingestion of fluid milk 14, and possibly by other dairy products, helping the sugar-induced insulin to expose the androgen receptor. But that is not milk's only contribution. It also contains absorbable insulin-like growth factor-1 (IGF-1) and milk ingestion also triggers endogenous elevations of IGF-1 15, a second signal to the phosphorylation and FoxO1-mediated androgen receptor exposure.

Having helped to over-expose the androgen receptors to the physiological load of androgens from ovaries, testes, adrenals, intracrine sources and the androgenic progestins in contraceptives 16, dairy adds its own complement of androgens and other DHT precursors 17. It is this complex mix of hormones, acting through the master growth regulator mTORC1, that leads to the increased production of intraductal keratinocytes that lies at the comedonal heart of both acne vulgaris and acne inversa. The concurrent and parallel (but arguably irrelevant) increase in sebocyte and sebum production responds to the same chain of stimuli.

The epiphenomena of Propionibacterium acnes overgrowth, Malassezia furfur multiplication, follicular wall rupture, inflammation both innate and adaptive, folliculopilosebaceous unit disintegration, secondary non-commensal bacterial infection, sinus formation and scarring are all downstream from mTORC1.

The sites of interaction with FoxO1 and mTORC1, and the *modus operandi,* of each of our present therapeutic weapons are detailed for the first time ever. Within this matrix, the arrows pointing to future therapeutic opportunities are posted. We will still need to employ active therapy for years to come because, once the fire is lit, extinguishing the match is not enough to put out the fire it started.

No doubt we have still more to learn, but this is a giant step forward. There have been 18, and likely will be more, conservative voices who insist on proof beyond doubt. Theirs will be a long wait. The design and conduct of a satisfactory prospective randomized clinical trial faces several unique challenges. It will require the recruitment of acne-prone individuals who do not yet have acne. They will need parents willing to have their children blindly consume real dairy products or counterfeits that have been tested as safe and healthy despite having been modified to remove all steroid hormones, growth hormones and other potential acnegens 19, without disturbing the taste and texture of the original. This is a tall order indeed, especially in the young and challenging population affected. Add into the equation the vast number of parents for whom dairy is beyond reproach as a perfect food. Top off the problem with a generation or two (or three) of physicians who believe the same, and one wonders if such a proposed study would ever pass through an institutional review board.

It is time to heed the words of Albert Kligman and strive ‘to actually achieve the ultimate goal in medical practice, namely prevention’ 20. Teaching the acne-prone to embrace a new dietary lifestyle is an uphill battle, even with the strength of Melnik's translational research behind us. It would be less so if we were all on side.
